# SARAF and Orai1 Contribute to Endothelial Cell Activation and Angiogenesis

**DOI:** 10.3389/fcell.2021.639952

**Published:** 2021-03-04

**Authors:** Isabel Galeano-Otero, Raquel Del Toro, Abdel-Majid Khatib, Juan Antonio Rosado, Antonio Ordóñez-Fernández, Tarik Smani

**Affiliations:** ^1^Department of Medical Physiology and Biophysics, University of Seville, Seville, Spain; ^2^Group of Cardiovascular Pathophysiology, Institute of Biomedicine of Seville, University Hospital of Virgen del Rocío/University of Seville/CSIC, Seville, Spain; ^3^CIBERCV, Madrid, Spain; ^4^LAMC, INSERM U1029, Pessac, France; ^5^Department of Physiology, University of Extremadura, Caceres, Spain; ^6^Department of Surgery, University of Seville, Seville, Spain

**Keywords:** Orai1, SARAF, SOCE, HUVEC, angiogenesis

## Abstract

Angiogenesis is a multistep process that controls endothelial cells (ECs) functioning to form new blood vessels from preexisting vascular beds. This process is tightly regulated by pro-angiogenic factors, such as vascular endothelial growth factor (VEGF), which promote signaling pathways involving the increase in the intracellular Ca^2+^ concentration ([Ca^2+^]_i_). Recent evidence suggests that store-operated calcium entry (SOCE) might play a role in angiogenesis. However, little is known regarding the role of SARAF, SOCE-associated regulatory factor, and Orai1, the pore-forming subunit of the store-operated calcium channel (SOCC), in angiogenesis. Here, we show that SOCE inhibition with GSK-7975A blocks aorta sprouting, as well as human umbilical vein endothelial cell (HUVEC) tube formation and migration. The intraperitoneal injection of GSK-7975A also delays the development of retinal vasculature assessed at postnatal day 6 in mice, since it reduces vessel length and the number of junctions, while it increases lacunarity. Moreover, we find that SARAF and Orai1 are involved in VEGF-mediated [Ca^2+^]_i_ increase, and their knockdown using siRNA impairs HUVEC tube formation, proliferation, and migration. Finally, immunostaining and *in situ* proximity ligation assays indicate that SARAF likely interacts with Orai1 in HUVECs. Therefore, these findings show for the first time a functional interaction between SARAF and Orai1 in ECs and highlight their essential role in different steps of the angiogenesis process.

## Introduction

Angiogenesis is defined as the formation of new blood vessels from existing vasculature for the purpose of expanding vascular networks to the tissues (Stapor et al., 2014). This process includes microvascular growth and endothelial sprouting, which itself involves endothelial cell (EC) proliferation, migration, and tube formation (Mentzer and Konerding, 2014). Angiogenesis displays fine-tuned regulation that is mainly stimulated by oxygen deficiency, which may happen in both physiological (e.g., reproduction (Logsdon et al., 2014) or tissue repair (Ingason et al., 2018)) and pathological situations (e.g., diabetic retinopathy (Li et al., 2011) or cancer (Folkman, 1971)). This process requires the action of several growth factors, including vascular endothelial growth factor (VEGF), considered as the most pro-angiogenic factor, and fibroblast growth factor (FGF) and epidermal growth factor (EGF), which are secreted by parenchymal cells and triggered by the hypoxic environment (Adair and Montani, 2010).

Vascular endothelial growth factor promotes angiogenesis by its binding to VEGF receptors, VEGFR1 and VEGFR2 (Dragoni et al., 2011). Previous studies demonstrated that VEGF addition to ECs mediates the classical intracellular Ca2 + release followed by extracellular Ca^2+^ entry (Faehling et al., 2002; Jho et al., 2005). VEGF-induced increase in the intracellular Ca^2+^ concentration ([Ca^2+^]_i_) was related to the activation of the store-operated calcium entry (SOCE) pathway (Li et al., 2011), which is required to the angiogenic activity in EC (Chen et al., 2016). Within the key elements of SOCE, Orai1, the pore-forming subunit of the store-operated calcium channel (SOCC), and stromal interacting molecule 1 (STIM1), are the most studied proteins (Avila-Medina et al., 2020). Both assemble to allow the activation of Ca^2+^ release-activated Ca Ca^2+^ (CRAC) channels, responsible for SOCE currents. Previous studies demonstrated that Orai1 and STIM1 are involved in the proliferation and CRAC currents of EC (Abdullaev et al., 2008). Likewise, it has been proved that siRNA-mediated inhibition of Orai1 and STIM1 affected angiogenesis *in vitro* using human umbilical vein EC (HUVEC) and endothelial progenitor cells (Lodola et al., 2012).

Recently, SARAF (SOCE-associated regulatory factor) has been proposed as a new regulator of STIM1 activation. SARAF blocks the spontaneous activation of STIM1 under resting conditions (Palty et al., 2012). Likewise, SARAF attenuates arachidonate-regulated channel (ARC) activity, constituted by subunits of Orai1 and Orai3 (Albarran et al., 2016b). Other studies showed that Orai1 is essential for the interaction between STIM1 and SARAF (Palty et al., 2012; Jardín et al., 2018). Interestingly, it has been demonstrated that SARAF and Orai1 work together to boost SOCE in highly proliferated cancers cells, MEG01 and NG115-401L, independently of STIM1 (Albarran et al., 2016a). Nevertheless, to the best of our knowledge the role of SARAF in angiogenesis has not been addressed. Therefore, in this study we investigate the role of SARAF and Orai1 using different angiogenic approaches.

## Materials and Methods

All animal assays were done in accordance with the recommendations of the Royal Decree 53/2013 in agreement with the Directive 2010/63/EU of the European Parliament and approved by the local Ethics Committee on human Research of the “Virgen del Rocio” University Hospital of Seville.

### Cell Culture and Transfection

Human umbilical vein endothelial cells (HUVEC; Lonza, Basilea, Switzerland) were cultured in 25-cm^2^ flasks with enriched Endothelial Growth Medium BulletKit-2 (EGM-2) and were incubated at 37°C at 5% CO_2_. Primary cultures were thawed following the recommended seeding density from cryopreservation. After 24 h, the growth medium was replaced to refresh the medium. HUVECs were cultured and used at passages 3–10. HUVECs were transfected at 70% confluence with 3 μl of 10 μM siRNAs of scramble, Orai1, or SARAF using Lipofectamine^®^ RNAiMAX Transfection Reagent following the manufacturer’s instructions. Scrambled siRNA has the same nucleotide composition as the input sequence that is used as negative control.

### Tube Formation Assay in μ-Slide Angiogenesis

We studied tube formation as described previously (DeCicco-Skinner et al., 2014). We used μ-Slide Angiogenesis ibiTreat 15 wells from ibidi^®^ following the instructions of the “Application Note 19: Tube Formation” available in the web of ibidi^®^. Briefly, 10 μl of Matrigel was added to each well. Immediately, μ-Slide was placed into the incubator to allow gel polymerization for 30 min. Next, 1.10^4^ HUVECs suspended in 50 μl of EGM-2 were added to each well of the μ-Slide and were incubated at 37°C in 5% CO_2_; 18 h after, pictures were taken using a phase-contrast inverted microscope Olympus IX-71 (×4, objective). Next, the supernatant was discarded and 50 μl serum-free medium was added with 6.25 μg/ml of Calcein-AM. μ-Slide was then incubated for 30 min at RT in the dark. To analyze siRNA-mediated inhibition assay, cells were transfected 24 h before experiments. To analyze SOCC inhibition by GSK-7975A, the drug or vehicle (DMSO) was added after cells seeding in μ-slide wells. Mesh formation was determined using Angiogenesis Analyzer for ImageJ (Gilles, 2020).

### Mouse Retinal Angiogenesis

For retinal angiogenesis assay, neonatal mice (SV129) were injected intraperitoneally with increasing concentrations of GSK-7975A (2.6, 4.0, 7.9, 15.9, and 31.8 mg/kg) dissolved in DMSO at postnatal day P3, P4, and P5. Pups were sacrificed at P6. After that, retinas were isolated as previously (Del Toro et al., 2010). With little modification, briefly, eyes were extracted from the orbit and were fixed in 4% paraformaldehyde (PFA) at RT for 30 min. Next, retina was isolated and incubated with the permeabilization and blocking solution 2 h at 4°C. After that, retinas were incubated at 4°C overnight with 1:50 biotinylated isolectin B4 (IB4). Next day, retina was incubated with 1:200 Cy3 streptavidin for 2 h at RT. Before mounting with Dako, retina was postfixed with PFA for 20 min. Fluorescence images were collected with fluorescence microscope Olympus BX-61 (×4 objective). AngioTool software (Zudaire et al., 2011) was used to evaluate different parameters of vessel formation, such as total number of vessels and number of junctions, and lacunarity. Lacunarity is an index that measures and describes the distribution of the sizes of gaps or lacunae within retinal vessels (Gould et al., 2011).

### Endothelial Cell Migration

HUVEC migration *in vitro* was evaluated by wound-healing assay (Rodriguez et al., 2005). Briefly, HUVECs were seed in a 6-well plate and were cultured upon reaching 90–95% confluence. Then, using a sterile 2–200-μl pipette tip, a scratch was done. Next, after washing with PBS 1×, 1 ml of EGM-2 was added to each well. For siRNA experiments, HUVECs were transfected 48 h before scratching at 70% confluence. To analyze SOCC inhibition by GSK-7975A, the drug or vehicle (DMSO) was added after scratching. Pictures were taken by an inverted phase-contrast microscope Olympus IX71 (×10 objective) immediately, 12 and 24 h after scratching. The cell-free area in the wound was measured using Fiji ImageJ (NIH; Bethesda, MD, United States).

### Cell Proliferation

HUVECs were seeded on coverslips, and cell transfection was done 48 hours before the assay. The cells were fixed with formalin, permeabilized with PBS 1 × 0.5% Triton X-100, and blocked with PBS 1× and 1% bovine serum albumin 0.5% TWEEN^®^ 20. HUVECs were then incubated with a mouse anti-Ki67 antibody for 2 h at room temperature (RT). After that, coverslips were incubated 45 min in the dark at RT with Goat anti-Mouse Alexa Fluor^®^ 594 (H + L) (1:200). HUVECs were incubated with DAPI diluted in PBS 1× during 5 min to visualize the nucleus. The coverslips were mounted using Dako. Cells were then photographed using an Olympus BX-61 fluorescence microscope (×10 objective). To analyze the proliferation, we considered Ki67^+^ or proliferating cells whose merge between the two channels matched (channel red: Ki67, channel blue: DAPI), using CellCounter of ImageJ (NIH; Bethesda, MD, United States).

### Spatial Co-localization Study by Immunofluorescence and Proximity Ligation Assay

In order to examine the co-localization of Orai1 and SARAF proteins, we performed immunofluorescence and *in situ* Proximity Ligation Assay (PLA). Briefly, HUVECs were seeded on coverslips, fixed with formalin, permeabilized with PBS 1 × 0.5% Triton X-100, and blocked with PBS 1 × 1% bovine serum albumin + 0.5% TWEEN^®^ 20. Cells were then incubated with mouse anti-Orai1 (1:200) and rabbit anti-SARAF (1:200) antibodies for 2 h at RT. After that, the coverslips were incubated 45 min in the dark at RT with Goat anti-mouse Alexa Fluor^®^ 594 (H + L) (1:400) and Goat anti-rabbit Alexa Fluor^®^ 488 (1:400). Staining with DAPI was used to visualize the nucleus of HUVEC. The coverslips were mounted using Dako. Cells were photographed by a Nikon A1R + laser scanning confocal microscope (×40 objective). The Pearson correlation coefficient (PCC) was calculated using Jacob plugin of ImageJ software (Bolte and Cordelières, 2006).

For PLA assay, we used the Duolink *in situ* PLA detection kit (Sigma-Aldrich, St Louis, MO, United States). HUVECs were seeded on ibidi μ-Slide VI^0.4^ ibitreat and fixed with formalin during 25 min following the manufacturer’s instruction. After blocking for 30 min, cells were incubated with mouse anti-Orai1 and rabbit anti-SARAF antibodies for 2 h at RT. After that, cells were incubated with Duolink PLA anti-rabbit PLUS and anti-mouse MINUS included in the kit during 1 h at 37°C. Next, for the ligation step, we added hybridized oligonucleotides with the ligase to cells and further incubated them for 30 min at 37°C. Cells were then incubated with the polymerase diluted in the amplification buffer for 100 min at 37°C. The three last steps were done in a preheated humidity chamber. HUVECs were then washed following the instructions and incubated with DAPI for nucleus visualization. Cells were photographed using the Olympus IX-71 fluorescence microscope (×20 objective).

### Aorta Ring Assay

Aorta ring assay was done following a modified protocol described by Baker *et al.* (Baker et al., 2012). Thoracic aortas were obtained from 250 to 300 g male Wistar rats. After dissection, aorta was sliced in 0.5-mm divisions. Then, rings were incubated in EGM-2 at 37°C overnight. Next day, 50 μl of Matrigel was added to wells of a 24-well plate which was incubated for 15 min at 37°C. Each aortic ring was collocated over the Matrigel drop, and 50 μl of Matrigel was added again to seal the ring. After 15 min of incubation at 37°C, we added 500 μl of EGM-2 with increasing concentrations (10, 30, 50, 70, and 100 μM) of GSK-7975A, or DMSO as vehicle. Photos were taken immediately and each 48 h until day 6, using a phase-contrast microscope Olympus IX-71 (×10, objective). Cell sprouting was evaluated using Fiji ImageJ.

### Intracellular Calcium Study

Ca^2+^ measurement was carried out in HUVECs loaded with 2 to 5 μM Fura-2 AM using an imagine system. The recording system consists of an inverted microscope Leica (Wetzlar, Germany) equipped with a 20×/0.75 NA objective, a monochromator (Polychrome V, Till Photonics, Munich, Germany), and a light-sensitive CCD camera, controlled by HP software (Hamamatsu Photonics, Japan). Changes in intracellular Ca^2+^ are represented as the ratio of Fura-2 AM fluorescence induced at an emission wavelength of 510 nm due to excitation at 340 and 380 nm (ratio = *F*_340_/*F*_380_). Experiments were done in free Ca^2+^ solution (in mM: 140 NaCl, 2.7 KCl, 4 MgCl_2_, 0.5 EGTA, 10 HEPES, pH = 7.4), and Ca^2+^ influx was determined from changes in Fura-2 fluorescence after re-addition of Ca^2+^ (2.5 mM). HUVECs were incubated 5 min with 30 ng/ml of VEGF and/without 30 ng/ml of anti-VEGF before Ca^2+^ addition; 10 μM of GSK-7975A was added before the end of the experiment. The Ca^2+^ influx (Δratio) was calculated as the difference between the peak ratio after extracellular Ca^2+^ re-addition and its level right before.

### RNA Isolation and Quantification

We used miRNeasy kit to extract RNAs from cells. Briefly, HUVECs were collected using 1 ml of QIAzol Lysis Reagent included in the kit and Cell Scrapers (Greiner Bio-One North America, Monroe, NC, United States). After mixing with 200 μl of chloroform, we followed the manufacturers’ instruction to get the eluted RNA. RNA was quantified using NanoDrop^TM^, and 1 μg of RNA was retro-transcribed into cDNA using iScript^TM^ Advanced cDNA Synthesis Kit. To determine genes’ expression, we used 2.5 μl of cDNA of each primer (Orai1, SARAF, 18S; Table 1), and 5 μl of iTaq Universal SYBR Green Supermix in a total volume of 10 μl of reaction. qRT-PCR was performed using an Applied Biosystems Viia7 7900HT thermocycler (Thermo Fisher Scientific, Waltham, MA, United States).

**TABLE 1:** Forward and reverse primers used to quantify the mRNA expression of Orai1, SARAF, and 18S.

**Primer**	**Sequence (5′–3′)**
Orai1	Forward: 5′-CCATAAGACGGACCGACAGT-3′ Reverse: 5′-GGGAAGGTGAGGACTTAGGC-3′
SARAF	Forward: 5′-CAGTGGGAATGTAAGACGGACTT-3′ Reverse: 5′-ACTCATAGCCTTCACAGCTCACC-3′
18S	Forward: 5′-AACGAGACTCTGGCATGCT-3′ Reverse: 5′-GCCACTTGTCCCTCTAAGA-3′

### Statistical Analysis

Analyses were performed with GraphPad (GraphPad Software, Inc.). The results are presented as the mean and standard error of the mean (SEM). All variables were normally distributed. We used ordinary one-way ANOVA, and we performed multiple comparisons using T test without correction (Fisher’s LSD test). To calculate the IC50 value in the dose–response inhibition analysis [log(inhibitor) vs. normalized responses], we used Hill equation, *Y* = 100/[1 + 10^{(logIC50 - log*X*)^∗^*n*}], where *n* is the Hill slope.

### Reagents

To culture HUVECs, we used Endothelial Growth Medium BulletKit-2 (EGM-2^TM^ BulletKit; Lonza, Basilea, Switzerland) enriched with EGM^TM^-2 SingleQuots^TM^ (2% FBS, hydrocortisone, hFGF-B, VEGF, R3-IGF-1, hEGF, ascorbic acid, gentamicin/ampicillin, and heparin). In order to transfect HUVECs, we used Lipofectamine^®^ RNAiMAX Transfection Reagent (Thermo Fisher Scientific, Waltham, MA, United States) and the siRNAs of scramble, Orai1, or SARAF (Ambion, Thermo Fisher Scientific, Waltham, MA, United States). SOCC inhibition was studied using GSK-7975A (Aobious, Gloucester, MA, United States) (Derler et al., 2013). The drug was dissolved in dimethyl sulfoxide (DMSO; Sigma-Aldrich, St Louis, MO, United States), considered as vehicle in a different set of experiments. VEGF (Sigma-Aldrich, St Louis, MO, United States), anti-VEGF (Cat. No. MAB293-SP; R&D, Minneapolis, MN, United States), and Fura-2 AM (Cat. No. F1225; Thermo Fisher Scientific, Waltham, MA, United States) were used for intracellular calcium study. Cell immunofluorescence permeabilization and blocking solution included PBS 1× with 0.5% Triton X-100 (Sigma-Aldrich, St Louis, MO, United States) and PBS 1× + 1% bovine serum albumin (BSA; Sigma-Aldrich, St Louis, MO, United States) and 0.5% TWEEN^®^ 20 (Sigma-Aldrich, St Louis, MO, United States), respectively. Mouse retina permeabilization and blocking solution included TNB blocking buffer [0.1 M Tris–HCl, pH 7.5; 0.15 M NaCl; 0.5% (w/v) blocking reagent from Perkin-Elmer] and 0.3% Triton X-100. We worked with these antibodies: mouse anti-Ki67 (1:50; Cat. No. 550609; BD Biosciences Pharmingen, San Diego, CA, United States), mouse anti-Orai1 (1:200; Cat. No. ab175040; Abcam, Cambridge, United Kingdom), rabbit anti-SARAF (1:200; Cat. No. PA5-24237; Thermo Fisher Scientific, Waltham, MA, United States), biotinylated isolectin B4 (IB4; 1:50; The Jackson Laboratory, Farmington, CO, United States), Goat anti-Mouse Alexa Fluor^®^ 594 (H + L) (Life technologies, Carlsbad, CA, United States), Goat anti-rabbit Alexa Fluor^®^ 488 (Life Technologies, Carlsbad, CA, United States), Cy3 streptavidin (The Jackson Laboratory, Farmington, CO, United States), and 4’,6-diamidino-2-phenylindole (DAPI; Sigma-Aldrich, St Louis, MO, United States). The coverslips were mounted using Dako Fluorescence Mounting medium (Dako; Agilent Technologies, Santa Clara, CA, United States). We used Corning^TM^ Matrigel^TM^ Matrix (Corning, NY, United States) to perform tube formation and rat aorta ring assays. To visualize live cells, we used Calcein-AM (Sigma-Aldrich, St Louis, MO, United States). To extract RNA from cells, we used miRNeasy kit (Qiagen, Hilden, Germany). RNA was retro-transcribed into cDNA using iScript^TM^ Advanced cDNA Synthesis Kit (Bio-Rad, Hercules, CA, United States) and quantified using iTaq Universal SYBR Green Supermix (Bio-Rad, Hercules, CA, United States). Primers of Orai1, SARAF, and 18S were purchased from Sigma (Sigma-Aldrich, St Louis, MO, United States).

## Results

### SOCC Inhibition With GSK-7975A Prevents Sprouting Angiogenesis, HUVEC Tube Formation, and Migration

**FIGURE 1:**
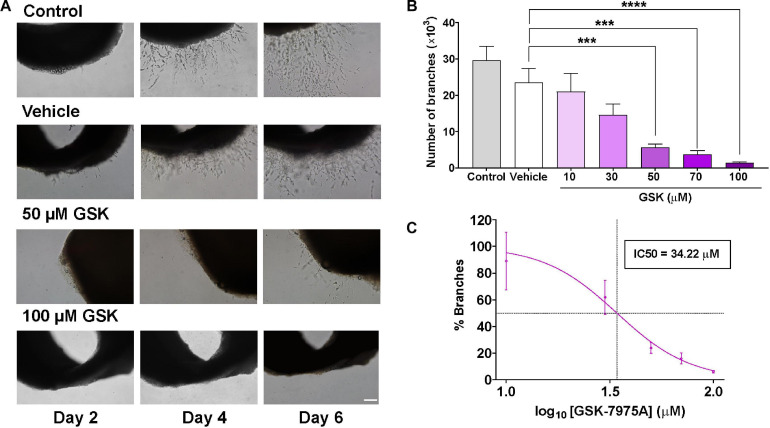
SOCC inhibition by GSK-7975A reduces aorta sprouting. Aorta was cultured in the endothelial cell culture medium (EGM-2) enriched with growth factors. **(A)** Phase-contrast imaging (×10 objective; scale bar = 200 μm) shows sprouting of aorta rings on day 2, 4, and 6 in untreated aorta (control), in aortic ring treated with DMSO (Vehicle), and in aortic rings incubated with GSK at 0, 50, and 100 μM. **(B)** Bar graph shows the number of branches in control rat aorta rings (gray) and in aorta treated with vehicle (DMSO; white) and with 10, 30, 50, 70, and 100 μM of GSK (purple) (*n* = 6). **(C)** Curve shows the dose-dependent inhibition of % branches mediated by GSK-7975A in the rat aorta ring assay. IC50 = 34.22 μM. The fit was done using Hill equation as described in Methods (Hill slope: −2.443, 95% CI IC50 22.86 to 44.85, *R*^2^ = 0.5762). Values are presented as means ± S.E.M. (***), and (****) indicate significance with *p* < 0.01, and *p* < 0.0001, respectively.

To examine the role of SOCC in angiogenesis, we used the *ex vivo* model of rat aorta ring assay to check whether the formation of microvessels can be affected by SOCC inhibition with GSK-7975A (GSK), a widely used SOCC inhibitor (Derler et al., 2013). As shown in Figure 1A, in control aorta rings embedded in Matrigel and immersed in endothelial cell culture medium (EGM-2) enriched with growth factors, the outgrowth of well-formed sprouts took place after 4 days in culture. Slightly fewer sprouts were observed in aortic ring incubated with 1% of DMSO (vehicle), although they were not significantly different than in control. By contrast, the addition of increasing concentrations of GSK prevented aortic sprouting. Data analysis in Figures 1B,C shows that the number of new branches diminished drastically in GSK-treated aortic rings in a dose-dependent manner with an IC50 of 34.22 μM, as compared to the vehicle group.

**FIGURE 2:**
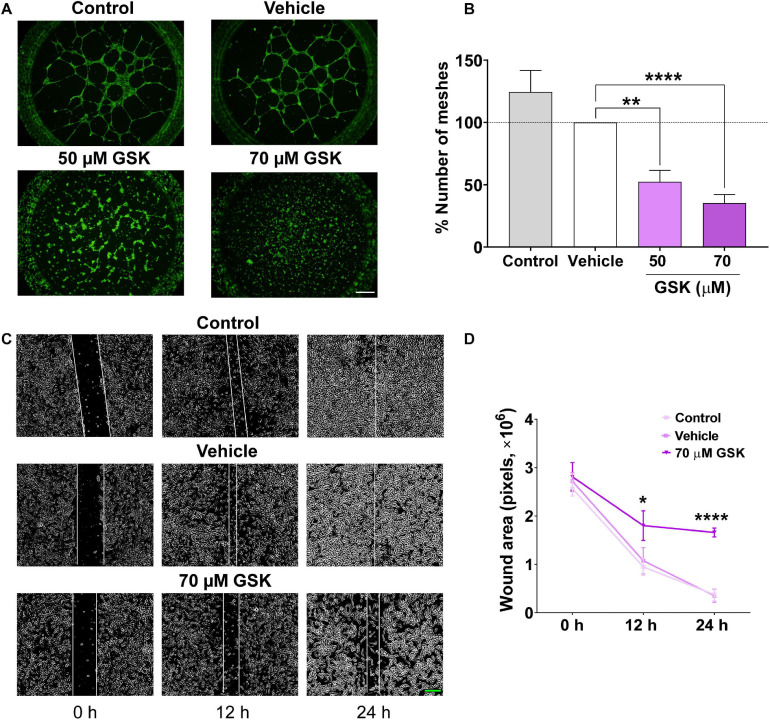
GSK-7975A decreases HUVEC tube formation and migration. **(A)** Fluorescence images (×4 objective; scale bar = 500 μm) are from HUVECs (control, vehicle, 50 and 70 μM of GSK) incubated with Calcein-AM and seeded on Matrigel. **(B)** Bar graph shows normalized means of the percentage of meshes to the number in HUVECs treated with vehicle (gray). Bars are for untreated HUVECs, for HUVECs treated with vehicle (white), and with 50 or 70 μM of GSK (*n* = 6). **(C)** Phase-contrast imaging (×10 objective; green scale bar = 200 μm) of the HUVEC wound healing assay. HUVEC was cultured in the endothelial cell culture medium (EGM-2) enriched with growth factors. Images are from control cells and for those treated with vehicle and 70 μM of GSK, taken at 0, 12, and 24 h. **(D)** Graph shows summary data of the evolution of the wound area (*n* = 6 per group). Values are presented as the means ± S.E.M. (*), (**), and (****) indicate significance with *p* < 0.05, *p* < 0.01, and *p* < 0.0001, respectively.

Next, we assessed the effect of GSK *in vitro*, using HUVEC-induced tube formation assay. As depicted in Figures 2A,B, the addition of GSK to HUVEC seeded on Matrigel resulted in a reduced capacity of HUVEC to align and form mesh-like structures. HUVEC preincubation with DMSO (vehicle) did not affect significantly the formation of meshes as compared to control. Furthermore, using a well-established wound healing assay, we observed in Figure 2C, after scratching HUVEC, both in control and vehicle groups, a significant reduction in the wound size during the first 12 h. In contrast, treatment of HUVEC with 70 μM GSK significantly attenuated cell migration, reaching the maximal significant effect 24 h after cell treatment (Figure 2D).

Altogether, these data indicate that pharmacological inhibition of SOCC with GSK impaired angiogenesis as assessed by tube formation, cell migration, and aorta ring assays.

### Intraperitoneal Injection of GSK-7975A Affects Retinal Angiogenesis

**FIGURE 3:**
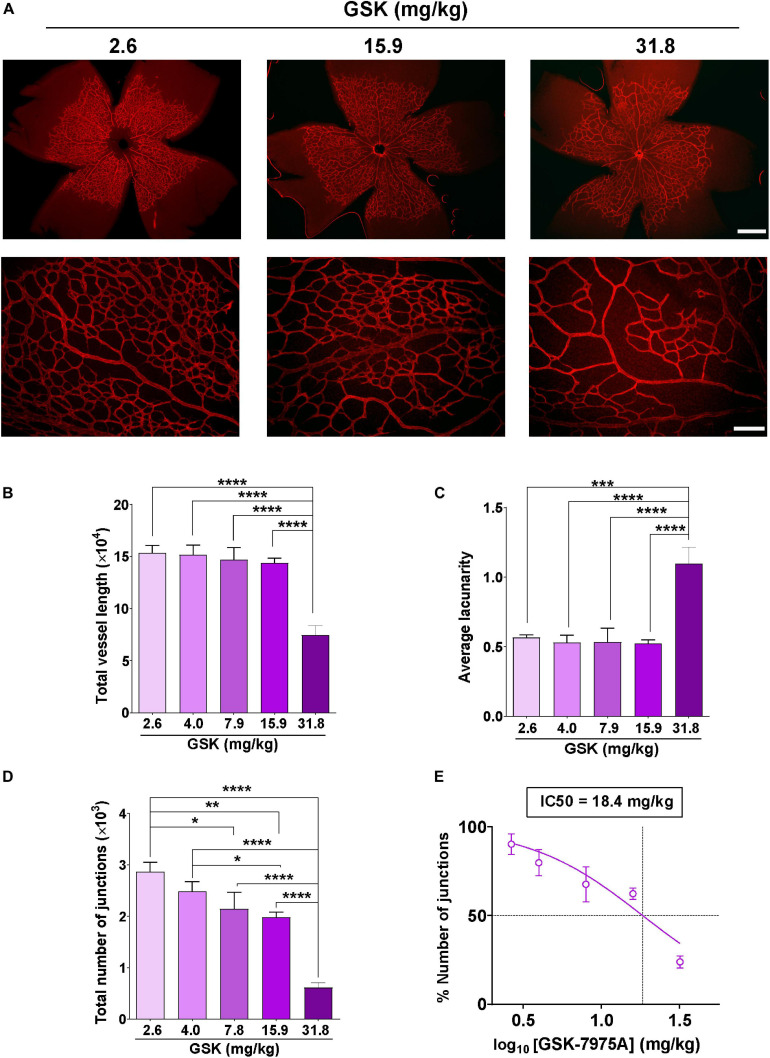
GSK-7975A alters the vascularization of the retina. **(A)** Representative images of retinal blood vessels stained with Isolectin B4. Retina was isolated from P6 mouse injected with 2.6, 15.9, and 31.8 mg/kg of GSK. Fluorescence images were taken with objectives ×4 (top; scale bar = 500 μm) and ×20 (bottom; scale bar = 100 μm). **(B–D)** Bar graphs show summary data of total vessel length **(B)**, the average of lacunarity **(C)**, and the total number of junctions **(D)** of mouse retina vessels injected with 2.6, 4.0, 7.9, 15.9, and 31.8 mg/kg of GSK (*n* = 4 to 8). **(E)** Curve shows the dose-dependent inhibition of % number of junctions affected by GSK-7975A IC50 = 18.4 mg/kg. The fit was done using the Hill equation (Hill slope: -1.185, 95% CI IC50 14.50 to 24.66, *R*^2^ = 0.6480). Values are presented as the means ± S.E.M. (*), (**), (***), and (****) indicate significance with *p* < 0.05, *p* < 0.01, *p* < 0.001, and *p* < 0.0001, respectively.

To further confirm the role of SOCC in angiogenesis, we evaluated the effect of intraperitoneal injection of GSK in retinal vascularization, using a mouse model of retinal angiogenesis (Del Toro et al., 2010). Increasing concentrations of GSK (from 2.6 to 31.8 mg/kg) were injected in neonatal mice at P3, P4, and P5, and retinal vessel formation was analyzed at P6. Figure 3A shows that vessel development was attenuated in the presence of increasing concentration of GSK. This delay in vessel formation was evident in the retina of mouse pups injected with 31.8 mg/kg GSK. AngioTool analysis determined that the total vessel length was significantly smaller when GSK was used at 31.8 mg/kg (Figure 3B) (Zudaire et al., 2011). Figure 3C shows that the maximum average of lacunarity, an index describing the distribution of the sizes of gaps between vessels, was also observed with 31.8 mg/kg GSK. In addition, the number of junctions decreased significantly with GSK concentrations higher than 4.0 mg/kg (Figure 3D). Figure 3E shows that in this case the effect of GSK was dose dependent with an IC50 value of 18.4 mg/kg. Supplementary Figure 1 in supporting information confirmed that all these parameters were significantly affected with 31.8 mg/kg GSK, as compare with the retina treated with the same amount of DMSO used as vehicle. These findings further confirm that the vascularization of retina can be affected by the SOCC inhibitor.

### Role of SARAF and Orai1 in VEGF-Induced Ca^2+^ Entry

**FIGURE 4:**
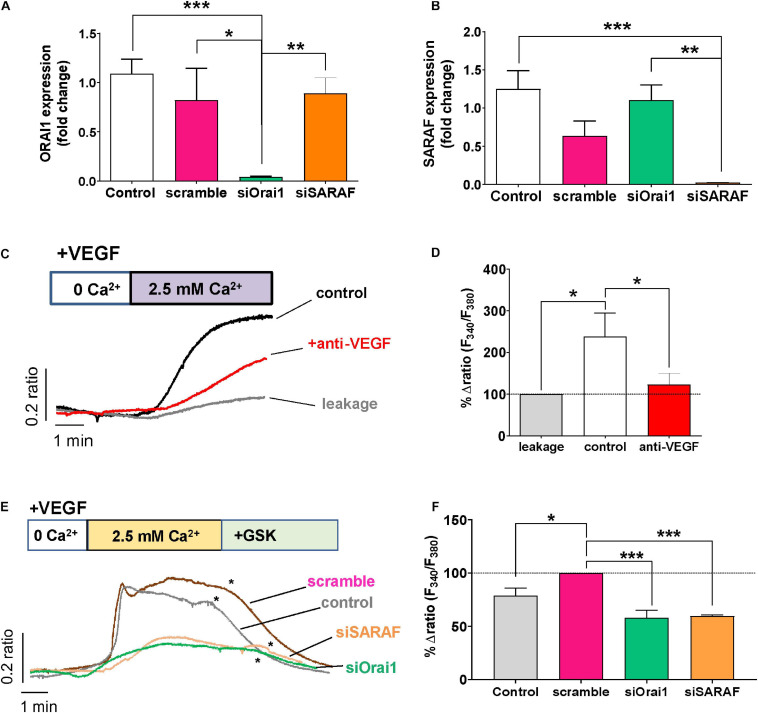
Role of Orai1 and SARAF in VEGF-induced Ca^2+^ entry. **(A,B)** Bar graphs show levels of mRNA expression (log fold change) of Orai1 **(A)** and SARAF **(B)** in control HUVECs (white, *n* = 8) and in cells transfected with scramble (pink, *n* = 4), siRNA Orai1 (green, *n* = 5), and siRNA SARAF (orange, *n* = 5). **(C)** Representative recordings of VEGF-induced changes in the [Ca^2+^]_i_ expressed as fluorescence ratio (F_340_/F_380_). HUVECs were incubated with 30 ng/ml VEGF (control) and with anti-VEGF (+ anti-VEGF) for 5 min in a free Ca^2+^ solution; 2.5 mM Ca^2+^ was re-added as indicated. Gray trace shows representative recording of non-specific Ca2 + entry in non-treated HUVECs after Ca^2+^ addition. **(D)** Bar graph shows the percentage of delta ratio increase after and before adding Ca^2+^ normalized to leakage (gray, *n* = 164) in cells treated with VEGF (white, *n* = 200) and in those incubated with VEGF + anti-VEGF (red, *n* = 255). **(E)** Representative recordings of VEGF (30 ng/ml)-induced changes in [Ca^2+^]_i_ in control HUVECs and those transfected with scramble siRNA or with Orai1 and SARAF siRNA. (*) indicates the addition of GSK-7975A (10 μM) at the end of each experiment. **(F)** Bar graph shows the percentage of delta ratio increase normalized to scramble (pink, *n* = 168 cells) after and before adding Ca^2+^ into non-transfected control cells (gray, *n* = 236 cells) and in cells transfected with siRNA Orai1 (green, *n* = 168 cells) and SARAF (orange, *n* = 115 cells). Values are presented as the means ± S.E.M. Significance is indicated by (*) for *p* < 0.05, (**) for *p* < 0.01, and (***) for *p* < 0.001.

To determine the role of the SOCE molecular component in angiogenesis, we used siRNA to examine the role of Orai1 and SARAF involvement in VEGF-mediated intracellular Ca^2+^ mobilization. As shown in Figures 4A,B, the transfection of HUVEC with siRNA of Orai1 and SARAF reduced drastically the expression of both Orai1 and SARAF mRNA; meanwhile, HUVEC transfection with scramble of RNA did not inhibit significantly the expression of Orai1 or SARAF, as compared to non-transfected control cells. Next, Figures 4C,D show that the re-addition of extracellular Ca^2+^ in HUVECs incubated with VEGF evoked a significant increase in [Ca^2+^]_i_. The induced [Ca^2+^]_i_ increase was significantly inhibited in HUVECs incubated both with VEGF and anti-VEGF. Furthermore, Figure 4E shows that VEGF stimulated a significant Ca^2+^ influx in cells transfected with scramble siRNA, which was slightly higher than in control non-transfected HUVEC. By contrast, VEGF-induced Ca^2+^ influx was significantly inhibited in HUVEC transfected with siRNA of Orai1 and SARAF. The addition of GSK at the end of each experiment successfully inhibited the Ca^2+^ influx, or what remained of this Ca^2+^ entry in transfected cells, confirming its SOCE nature. As depicted in Figure 4F, the downregulation of Orai1 and SARAF decreased VEGF-elicited Ca^2+^ response almost by 50%, as compared to cells transfected with scramble.

### Orai1 and SARAF Participate in HUVEC Tube Formation, Proliferation, and Migration

**FIGURE 5:**
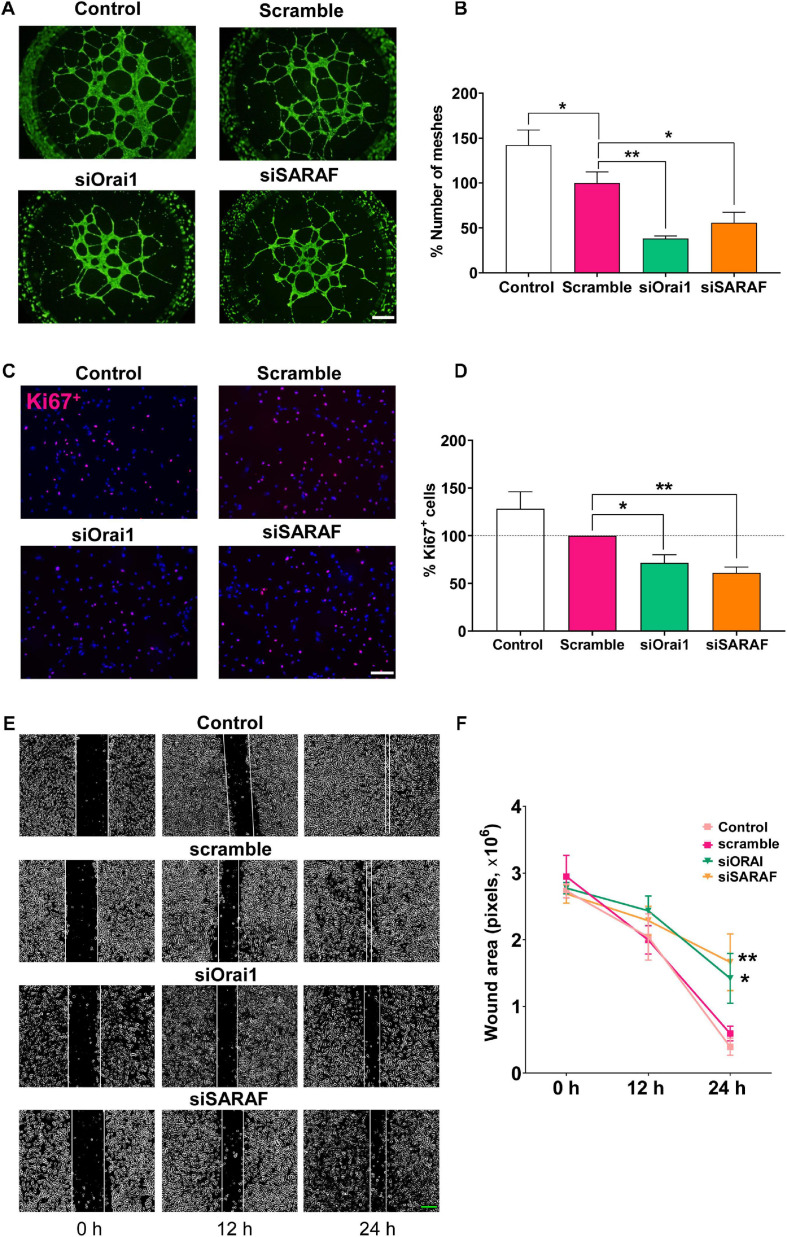
siRNA-mediated inhibition of Orai1 and SARAF attenuates HUVEC tube formation, proliferation, and migration. **(A)** Fluorescence images (×4 objective; scale bar = 500 μm) and **(B)** summary data (% of meshes number normalized to scramble) obtained from HUVECs embedded on Matrigel and stained with Calcein-AM of control (white) and transfected with scramble (pink), siRNA Orai1 (green), and siRNA SARAF (orange) (*n* = 5 to 6). **(C)** Merged representative images (×20 objective; scale bar = 100 μm) of HUVECs stained with Ki67^+^ (red) and DAPI (blue) in control and in cells transfected with scramble, or siRNA Orai1 and SARAF (*n* = 5). **(D)** Bar graph shows the percentage of Ki67^+^ control (white bar) and transfected HUVECs with siOrai1 and siSARAF, normalized to scramble. **(E)** Phase-contrast imaging (×10 objective; scale bar = 200 μm) of the HUVEC wound healing assay modified with ImageJ. Images were taken at 0, 12, and 24 h after the scratch from control HUVECs and form cells transfected with scramble siRNA, and siRNA against Orai1 and SARAF. **(F)** Graph shows summary data of the evolution of the wound area in experiments as in **(E)** (*n* = 4). Values are presented as the means ± S.E.M. Significance is indicated by (*) and (**) for *p* < 0.05 and *p* < 0.01, respectively.

The tube formation, migration, and proliferation of EC are considered critical early steps in the initiation of angiogenesis. Thereby, we examined the role of Orai1 and SARAF in these processes. As illustrated in Figure 5A, we observed that the transfection of HUVEC with siRNA against Orai1 and SARAF, seeded on Matrigel, prevented HUVEC capacity to mediate tube formation. Figure 5B indicates that Orai1 and SARAF silencing reduced mesh-like structures by approximately 60 and 40%, respectively, as compared to scramble. We also observed significantly less mesh formation in HUVECs transfected with scramble siRNA, as compared to control. Furthermore, Figures 5C,D illustrate that the incubation of HUVEC with EGM-2 enriched with growth factors promoted nucleus staining with Ki67 in control and cells transfected with scramble, indicating HUVEC proliferation. Conversely, Orai1 and SARAF downregulation by siRNA attenuated the amount of Ki67-positive HUVEC by 30 and 40%, respectively. We next evaluated cell migration using the wound-healing assay. Figures 5E,F indicate that while control HUVECs and those transfected with siRNA scramble closed the wound within 24 h, HUVECs transfected with siRNA of Orai1 and SARAF inefficiently sealed the wound over the same time frame. Therefore, these data demonstrated that Orai1 and SARAF are required for HUVEC tube formation, proliferation, and migration.

### Orai1 and SARAF Colocalize in HUVECs

**FIGURE 6:**
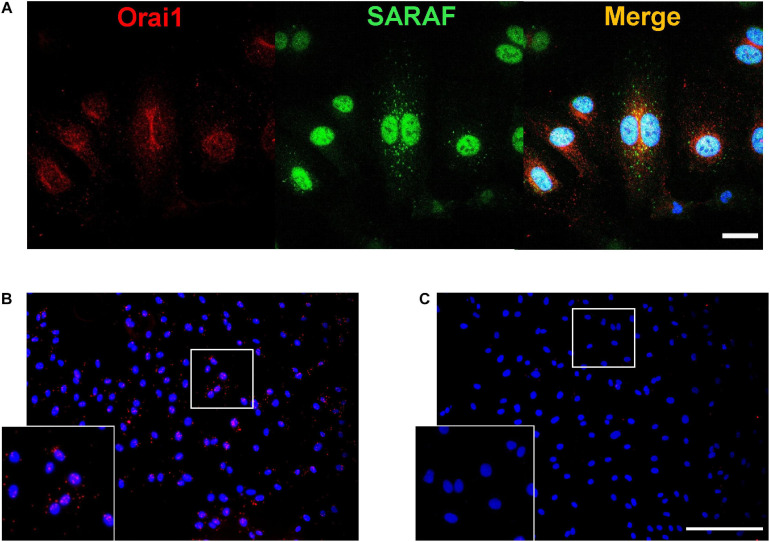
SARAF and Orai1 colocalization in HUVECs. **(A)** Representative images with immunofluorescence (×40 objective with ×2 zoom; scale bar = 25 μm) using specific antibodies show localization of SARAF (green) and Orai1 (red) in HUVECs stained with anti-rabbit SARAF and anti-mouse Orai1. Blue channel corresponds to DAPI. Merge image shows possible colocalization of SARAF with Orai1 as indicated by yellow color. **(B,C)** Representative images of fluorescence (20×; scale bar = 100 μm) in HUVECs using primary antibodies against Orai1 and SARAF (**B:** both; **C:** only Orai1) conjugated with the appropriate proximity ligation assay (PLA) probes. The bottom box is a zoom of (**B** and **C**) original images (20×). Red puncta indicate that proteins are in close proximity (<40 nm). HUVECs were cultured in the endothelial cell culture medium (EGM-2) enriched with growth factors, and nuclei are shown in blue as stained by DAPI.

Since our previous results strongly suggest a co-activation of Orai1 and SARAF in angiogenesis, we further examined the endogenous localizations of these proteins in HUVEC. Immunofluorescence images and analysis shows in Figure 6A that SARAF and Orai1 are uniformly distributed in HUVEC. Merge image and Pearson’s correlation coefficient (PCC), which estimate the correlation ratio, suggested a possible colocalization of SARAF and Orai1 in HUVECs since PCC was near 0.5 (*r* = 0.46). Then, we used proximity ligation assay (PLA) technique and we determined 644 of red puncta in 165 cells in HUVECs when incubated with primary antibodies against SARAF and Orai1 (Figure 6B), indicating that both proteins are in close proximity (<40 nm). Figure 6C shows that no PLA signal was detected in HUVEC conjugated with anti-Orai1 antibody, but without anti-SARAF antibody.

## Discussion

Angiogenesis is a dynamic multiphase process that includes the formation of new vessels from preexisting vascular beds, involving EC proliferation and migration, vascular patterning, and a final remodeling phase that ends in a stabilization of the new network for blood circulation (Ucuzian et al., 2010). The process of angiogenesis is tightly regulated by pro-angiogenic factors such as VEGF which binds to its receptors on ECs and mediates [Ca^2+^]_i_ increase (Munaron et al., 2008). The close relationship between EC physiology and Ca^2+^ signaling has been extensively studied (Filippini et al., 2019). Nevertheless, only few studies reported the role of SOCE in angiogenesis. In the current study, we described that SOCE activation plays a key role in several angiogenesis hallmarks, such as EC proliferation and migration, vessel sprouting, and tube formation. In fact, we demonstrated that GSK-7975A, a selective blocker of the CRAC channel (Derler et al., 2013), efficiently prevented aorta sprouting as well as HUVEC tube formation and migration. We also showed for the first time that intraperitoneal injection of GSK-7975A delayed the development of the retinal vasculature assessed at postnatal day 6 in mice. GSK-7975A reduced vessel length and their number of junctions, while it increased lacunarity, suggesting that SOCE might be required for normal vessel development. GSK-7975A inhibited especially and dose-dependently the number of branch junctions an essential step for vessel maturation and interconnected network formation, which may be independent of the number of new formed vessels and gaps that exist within them. The observed pharmacological inhibition of SOCE is consistent with previous studies which demonstrated that other more or less specific inhibitors of SOCE, Synta66 (Li et al., 2011), SFK-96365 (Chen et al., 2011; Savage et al., 2019), or 2-APB (Chen et al., 2011; Pafumi et al., 2015; Ye et al., 2018), similarly attenuated vessel formation in different *in vitro* and *in vivo* models of angiogenesis. Of note, none of these blockers have been injected to evaluate their effect on physiological developmental angiogenesis.

Within the key proteins of SOCE in the last years, Orai1 emerged as a possible new target to control angiogenesis, especially in tumor vascularization (as reviewed in Vashisht et al., 2015). Our data agree with previous studies that used VEGF or thapsigargin, the inhibitor of sarco/endoplasmic reticulum Ca^2+^-ATPase, to demonstrate the activation of SOCE and CRAC current in ECs. Abdullaev et al. (2008) showed that Orai1 knockdown also inhibited the proliferation of HUVEC. Likely, Li et al. (2011) used siRNAs, a dominant negative, or neutralizing antibodies to demonstrate that Orai1 is also necessary for HUVEC tube formation, as well as for VEGF-induced Ca^2+^ influx. In contrast, a previous study by Antigny et al. (2012) suggested that silencing of Orai1 did not affect tube formation when they used the EA.hy926 EC cell line; meanwhile, they suggest that STIM1, TRPC3, TRPC4, and TRPC5 are involved in this process (Antigny et al., 2012). However, in another study, Antigny et al. (2011) proposed that thapsigargin activated STIM1- and Orai1-dependent [Ca^2+^]_i_ increase in the same EC cell line. Our results support the involvement of Orai1 in HUVEC migration, proliferation, and Matrigel-based tube formation assays. At the same time, we demonstrated that VEGF-induced [Ca^2+^]_i_ increase was significantly attenuated following Orai1 and SARAF downregulation, confirming that VEGF triggers SOCE in HUVEC. HUVEC treatment by VEGF may activate other Ca^2+^-conducting channels since silencing of Orai1 and SARAF did not block completely the induced [Ca^2+^]_i_ increase, as reviewed elsewhere (Smani et al., 2018).

To the best of our knowledge, this study is the first to evaluate the participation of SARAF in angiogenesis. The role of SARAF, product of the *tmem*66 gene, in SOCE was described for the first time in 2012 (Palty et al., 2012). Now, there is a growing body of evidences indicating its role in the regulation of Ca^2+^ homeostasis in excitable and non-excitable cells (Albarran et al., 2016b). SARAF was identified as a blocker of spontaneous STIM1 activation under resting conditions in HEK cells (Palty et al., 2012). Later on, SARAF was demonstrated to regulate Orai1 activation through its binding to the STIM1 Orai1 activation region (SOAR) (Jha et al., 2013). Furthermore, Albarran et al. (2016b) found that SARAF is also expressed in the plasma membrane where it constitutively interacts with Orai1 and modulates Ca^2+^ entry through ARC (arachidonic acid regulated Ca^2+^) channels in neuroblastoma cell lines SH-SY5Y and NG115-401L. In the current study, using *in situ* PLA and immunofluorescence assays we demonstrated that Orai1 and SARAF are distributed in close subcellular vicinity suggesting their interaction. Indeed, Pearson’s correlation coefficient and the PLA puncta’s signal that occurs when proteins are < 40 nm apart (Bagchi et al., 2015) confirmed a strong colocalization of Orai1 and SARAF. This finding agrees with a previous data which demonstrated that SARAF colocalizes with STIM1 and Orai1, where it regulates the interaction between STIM1 and Orai1 during the initial steps of the activation of SOCE and transiently dissociates from STIM1 to associate with the C-terminus of Orai1 to promote Ca^2+^ entry (Albarran et al., 2016a).

Altogether, our data indicate that SARAF and Orai1 likely collaborate to maintain the Ca^2+^ influx required for different steps of angiogenesis. We provided the first evidence of SARAF expression in HUVEC, which interacts with Orai1 to sustain SOCE, HUVEC proliferation, migration, and tube formation. These findings suggest that SARAF and Orai1 may be good candidates to target angiogenesis in both physiological and pathological processes, such as cancer.

## Data Availability Statement

The original contributions presented in the study are included in the article/Supplementary Material, further inquiries can be directed to the corresponding author/s.

## Ethics Statement

The animal study was reviewed and approved by the Ethics Committee on Human Research of the “Virgen del Rocio” University Hospital of Seville.

## Author Contributions

IG-O, RD, JR, and TS contributed to the study conceptualization. IG-O, RD, and A-MK contributed to the study methodology. IG-O and TS contributed to the writing—original draft preparation. JR, AO-F, A-MK, and RD contributed to the writing—review and editing. RD and TS contributed to the supervision. TS contributed to the project administration. JR, AO-F, and TS contributed to the funding acquisition. All authors have read and agreed to the published version of the manuscript.

## Conflict of Interest

The authors declare that the research was conducted in the absence of any commercial or financial relationships that could be construed as a potential conflict of interest.

## Abbreviations

ARC: arachidonate-regulated channels
[Ca^2+^]_I_: intracellular Ca^2+^ concentration
CRAC: Ca^2+^ release-activated Ca^2+^
EC: endothelial cells
EGF: epidermal growth factor
EGM-2 ^TM^: Endothelial Growth Medium BulletKit-2
FGF: fibroblast growth factor
GSK: GSK-7975A
HUVEC: human umbilical vein EC
SARAF: SOCE-associated regulatory factor
SOAR: STIM1 Orai1 activation region
SOCE: store-operated calcium entry
SOCC: store-operated calcium channels
STIM1: stromal interacting molecule 1
VEGF: vascular endothelial growth factor.
