# Corrigendum: SARAF and Orai1 Contribute to Endothelial Cell Activation and Angiogenesis

**DOI:** 10.3389/fcell.2021.683097

**Published:** 2021-04-20

**Authors:** Isabel Galeano-Otero, Raquel del-Toro, Abdel-Majid Khatib, Juan Antonio Rosado, Antonio Ordóñez-Fernández, Tarik Smani

**Affiliations:** ^1^Department of Medical Physiology and Biophysics, University of Seville, Seville, Spain; ^2^Group of Cardiovascular Pathophysiology, Institute of Biomedicine of Seville, University Hospital of Virgen del Rocío/University of Seville/CSIC, Seville, Spain; ^3^CIBERCV, Madrid, Spain; ^4^LAMC, INSERM U1029, Pessac, France; ^5^Department of Physiology, University of Extremadura, Caceres, Spain; ^6^Department of Surgery, University of Seville, Seville, Spain

**Keywords:** Orai1, SARAF, SOCE, HUVEC, angiogenesis

There is an error in the Funding statement. The correct funding statement is: “This research was funded by Agencia Estatal de Investigación [PID2019-104084GB-C22/AEI/10.13039/501100011033]”.

The authors apologize for this error and state that this does not change the scientific conclusions of the article in any way. The original article has been updated.

