# Supplementary material for: SARAF and Orai1 Contribute to Endothelial Cell Activation and Angiogenesis
Source: Front Cell Dev Biol. 2021 Mar 4;9:639952. doi: 10.3389/fcell.2021.639952 (PMC7970240; doi:10.3389/fcell.2021.639952)
Supplement: Supplementary file 1 [file Image_1.pdf]

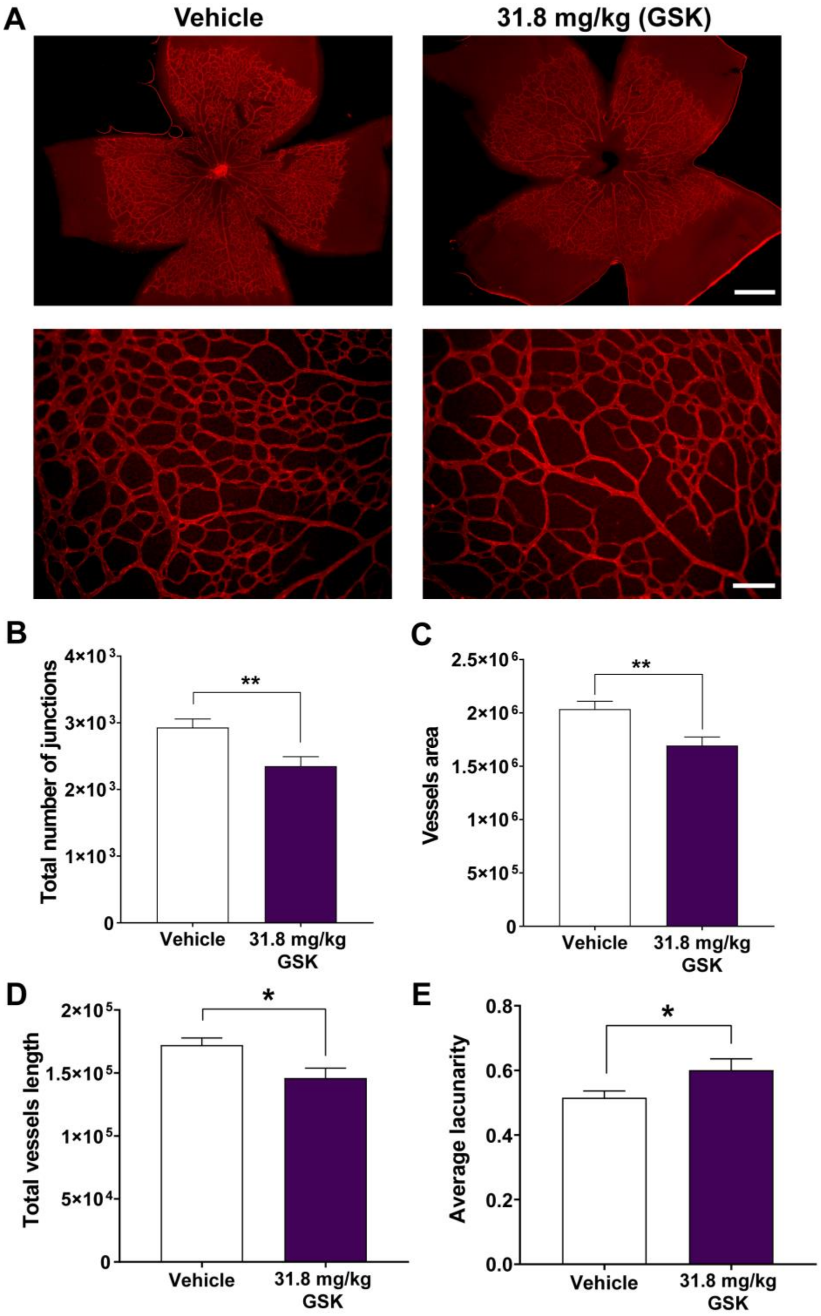

Supplementary Figure 1. GSK-7975A affects the retinal vascularization. (A) Representative image of retinal blood vessels stained with Isolectin B4. Retina was isolated from P6 mouse injected with 0 (vehicle: DMSO) or 31.8 mg/kg GSK. Fluorescence images were taken with objectives X4 (top; scale bar = 500 µm) and X20 (bottom; scale bar = 100 µm). Bar graphs show summary data of total number of junction (B), vessels area (C), total vessels length (D) and average lacunarity (E) of the mouse retina vessels injected with DMSO (n = 10) and 4.8 mM (n = 9) of GSK. Values are presented as the means ± SEM. Significance is indicated by (*) and (**) for $p<0.05$ and $p<0.01$, respectively.
