# Supplementary figures and images for: SARAF and Orai1 Contribute to Endothelial Cell Activation and Angiogenesis
Source: Front Cell Dev Biol. 2021 Mar 4;9:639952. doi: 10.3389/fcell.2021.639952 (PMC7970240; doi:10.3389/fcell.2021.639952)

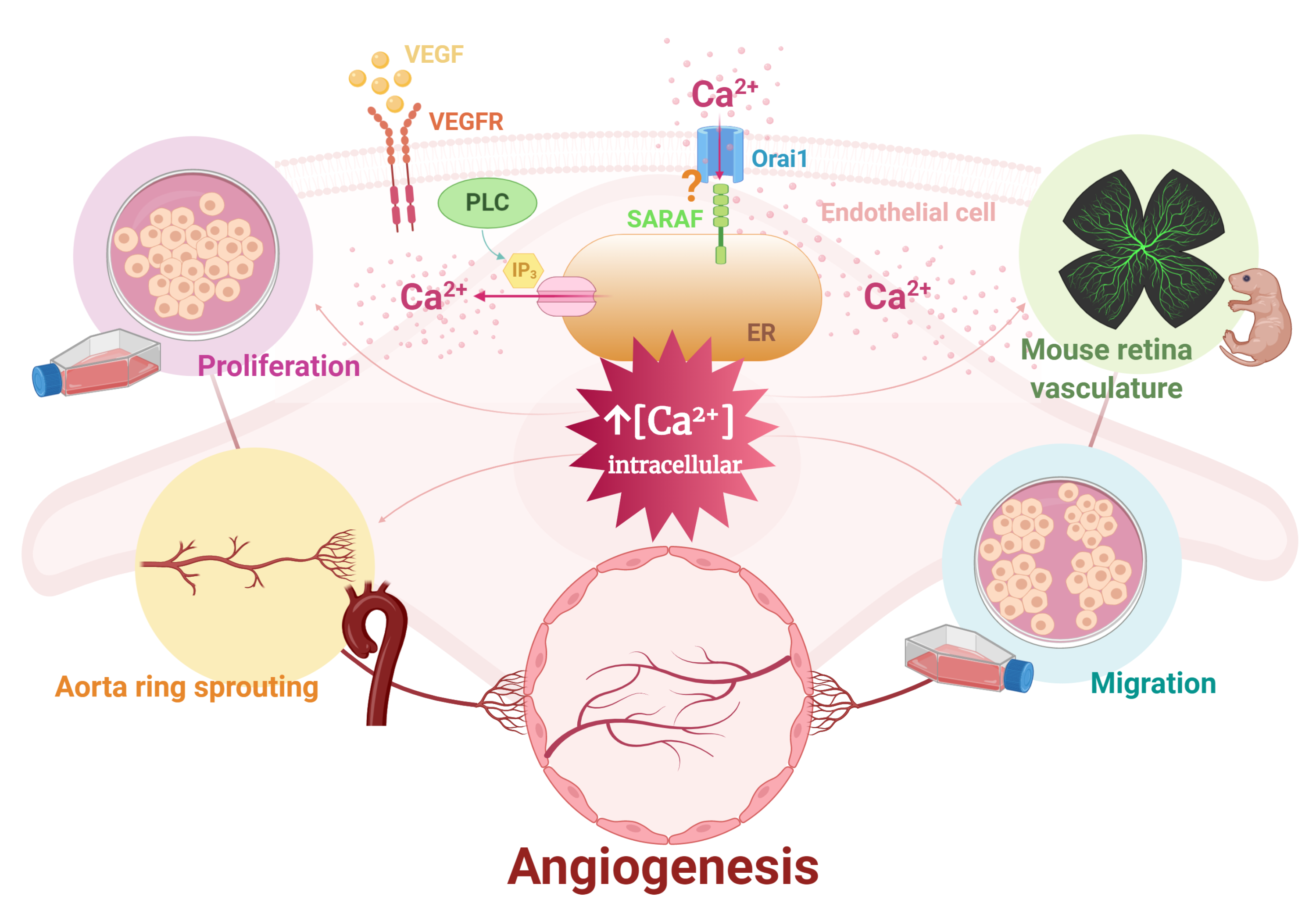

Supplement: Supplementary file 2 [file Image_2.PNG]
